# Vulnerability of Brazilian municipalities to hantavirus infections based on multi-criteria decision analysis

**DOI:** 10.1186/s12982-015-0036-5

**Published:** 2015-10-01

**Authors:** Stefan Vilges de Oliveira, Lidsy Ximenes Fonseca, Keline Medeiros de Araújo Vilges, Fernanda Voietta Pinna Maniglia, Simone Valéria Costa Pereira, Eduardo Pacheco de Caldas, Pedro Luiz Tauil, Rodrigo Gurgel-Gonçalves

**Affiliations:** Programa de Medicina Tropical, Faculdade de Medicina, Universidade de Brasília, Brasília, Brazil; Coordenação Geral de Doenças Transmissíveis, Secretaria de Vigilância em Saúde, Ministério da Saúde, Brasília, Brazil; Laboratório de Parasitologia Médica e Biologia de Vetores, Faculdade de Medicina, Universidade de Brasília, Brasília, Brazil

**Keywords:** Projections and predictions, Hantavirus, Epidemiology

## Abstract

**Background:**

Hantavirus infection is an emerging zoonosis transmitted by wild rodents. In Brazil, high case-fatality rates among humans infected with hantavirus are of serious concern to public health authorities. Appropriate preventive measures partly depend on reliable knowledge about the geographical distribution of this disease.

**Methods:**

Incidence of hantavirus infections in Brazil (1993–2013) was analyzed. Epidemiological, socioeconomic, and demographic indicators were also used to classify cities’ vulnerability to disease by means of multi-criteria decision analysis (MCDA).

**Results:**

From 1993 to 2013, 1752 cases of hantavirus were registered in 16 Brazilian states. The highest incidence of hantavirus was observed in the states of Mato Grosso (0.57/100,000) and Santa Catarina (0.13/100,000). Based on MCDA analysis, municipalities in the southern, southeastern, and midwestern regions of Brazil can be classified as highly vulnerable. Most municipalities in northern and northeastern Brazil were classified as having low vulnerability to hantavirus cardiopulmonary syndrome.

**Conclusions:**

Although most human infections by hantavirus registered in Brazil occurred in the southern region of the country, a greater vulnerability to hantavirus was found in the Brazilian Midwest. This result reflects the need to strengthen surveillance where the disease has thus far gone unreported.

## Background

Hantavirus infection is an emerging a zoonosis produced by *Bunyaviridae* viruses (genus *Hantavirus*). It is the cause of two distinct syndromes: hemorrhagic fever with renal syndrome (HFRS), which is endemic to Europe and Asia; and hantavirus cardiopulmonary syndrome (HCPS), which is restricted to the Americas [1]. HCPS is an emerging disease mainly transmitted by wild rodents (Rodentia: Cricetidae: Sigmodontinae) [2]. However, hantavirus infection has been described in insectivore mammal families, including Soricidae, Talpidae, Solenodontidae, and Nesophontidae. Hantavirus has also been detected in bats [3].

**Fig. 1:**
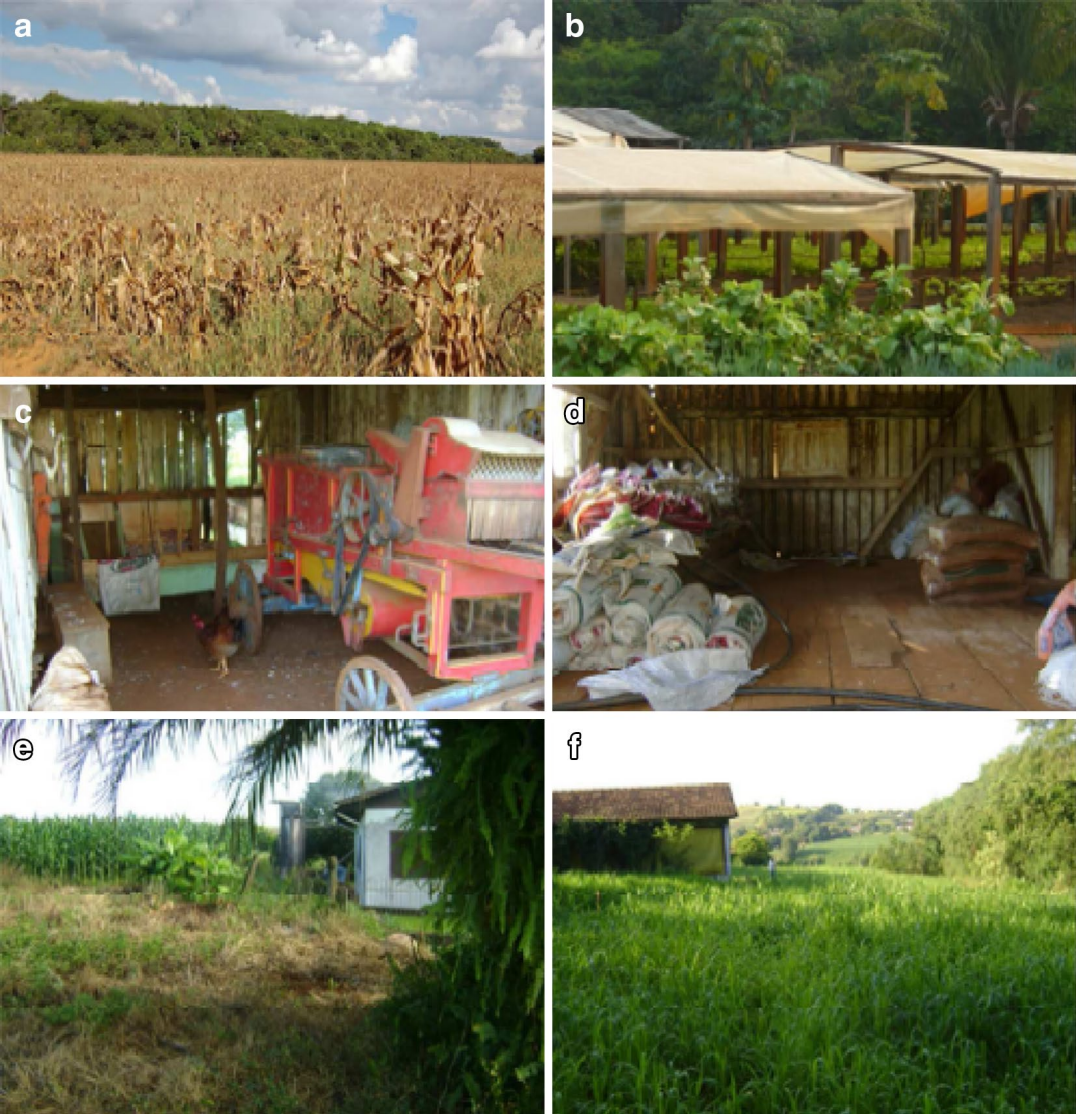
Main risk factors for hantavirus infections in Brazil. **a**, **b** Expansion of agricultural activities (corn and vegetables respectively); **c**, **d** domestic activities associated with exposure to rodents (livestock and grain storage, respectively); **e**, **f** houses near forest remnants where wild rodents occur

Hantavirus is currently registered all over Brazil. The high case-fatality rate of HCPS (~40 %) is of great concern to Brazilian public health authorities. Most patients require intensive health care [4], and case-fatality rates vary among Brazil’s five geographic regions [4–6]. The differences may be associated with circulating hantavirus strains in Brazil [7]. The risk factors for hantavirus infections include involvement in agricultural, domestic, and leisure activities (Fig. 1), which are associated with human exposure to rodents or their excreta [8].

The first Brazilian cases of HCPS were reported in 1993. These cases were associated with massive rodent infestation, which itself was linked to deforestation and the establishment of precarious households [9]. Poor living and housing conditions in rural areas are also strongly associated with hantavirus transmission in Brazil [8]. Buildings in substandard conditions allow for the invasion of wild rodents, which are attracted to stored food [10]. Other factors may also contribute to the occurrence of hantavirus: (1) rodent outbreaks associated with bamboo flowering; (2) improper environmental management; (3) urban informal housing settlements; and (4) ecosystem changes due to the construction of roads and hydroelectric dams [4, 6, 8, 11–18].

In the current study, the incidence of hantavirus infections in Brazil over the past 20 years (1993–2013) was analyzed. In addition, epidemiological, socioeconomic, and demographic indicators were used in multi-criteria decision analysis to classify vulnerability to disease in municipalities.

## Methods

### HCPS data

Cases of hantavirus were confirmed by laboratory tests, including serology for specific IgM antibodies (ELISA with recombinant antigens from hantavirus strains identified in the country), immunohistochemistry, and RT-PCR for hantavirus. Most of cases of hantavirus registered in our database were confirmed by laboratory tests. Some cases were confirmed using clinical and epidemiological criteria, as in the cases of individuals with acute respiratory failure who had been exposed to the same risk factors as the individuals in the laboratory-confirmed cases [8]. Occurrence data on confirmed HCPS cases reported to the Brazilian Ministry of Health between 1993 and 2013 were included in the study. Data were obtained from the Brazilian National Notifiable Diseases Surveillance System (SINAN) [8]. Descriptive analyses were based on demographic and epidemiological variables from investigation questionnaires. Healthcare professionals were responsible for filling out these forms. The questionnaires included clinical, epidemiological, and laboratory data. All confirmed cases of hantavirus in the municipalities that were the likely site of infection were counted. Average coefficients of incidence of hantavirus infection were calculated for municipalities, states, and the five Brazilian geopolitical regions (cases/100,000 inhabitants per year).

### Socioeconomic and demographic data

Socioeconomic and demographic indicators were obtained from Brazilian censuses conducted by the Brazilian Institute of Geography and Statistics, or IBGE (http://www.ibge.gov.br).

The municipality indicators used were economic gains from agriculture, or EGA (2006), the municipal human development index, or MHDI (2008), and the degree of urbanization, or DU (2008). We selected EGA to represent farming production in each municipality, since farming production is thought to be associated with human exposure to wild rodents [12]. The MHDI was selected because other studies have shown that human exposure to hantavirus is also linked to low levels of human development and poverty [1, 4, 8, 13, 15]. Finally, DU was selected because some studies have shown an association between urbanization and hantavirus occurrence [12, 16].

### Multi-criteria decision analysis (MCDA)

Assessment of municipalities’ vulnerability to hantavirus infection is a multi-criteria decision problem [20]. MCDA has been used to classify areas as vulnerable to *Trypanosoma cruzi* transmission in the USA [21] and in Brazil [19]. This method is based on the ranking of values and is often used for decision making after the consideration of multiple aspects of descending importance, organized into a hierarchy [22, 23].

A free application known as the program to support decision making based on indicators (PRADIN), version 3.0 (http://www.anipes.org.br) was used for the MCDA. This application implements the Preference Ranking Organization Method for Enrichment of Evaluations (PROMETHEE II) algorithm [22] from routines written in Visual Basic 6.0, as presented by Gomes [23]. MCDA has been applied with greater frequency in Brazil for the spatial identification of vulnerable areas, for health program monitoring, and to provide support for decision making based on indicators [24–26]. The importance assigned to the indicators used in the ranking was based on specific criteria: epidemiological indicators (hantavirus incidence) received a higher weight [4], and the others (economic gains from agriculture, municipal human development index, and degree of urbanization) received equal weights [2] in the MCDA analysis.

After their ranking, the municipalities were classified into quintiles. The geographic coordinates of the municipalities (http://www.ibge.gov.br) were used in the Tabwin 32 program (http://www2.datasus.gov.br/DATASUS/index) to produce vulnerability maps showing the occurrence of hantavirus infections in the country.

## Results

**Fig. 2:**
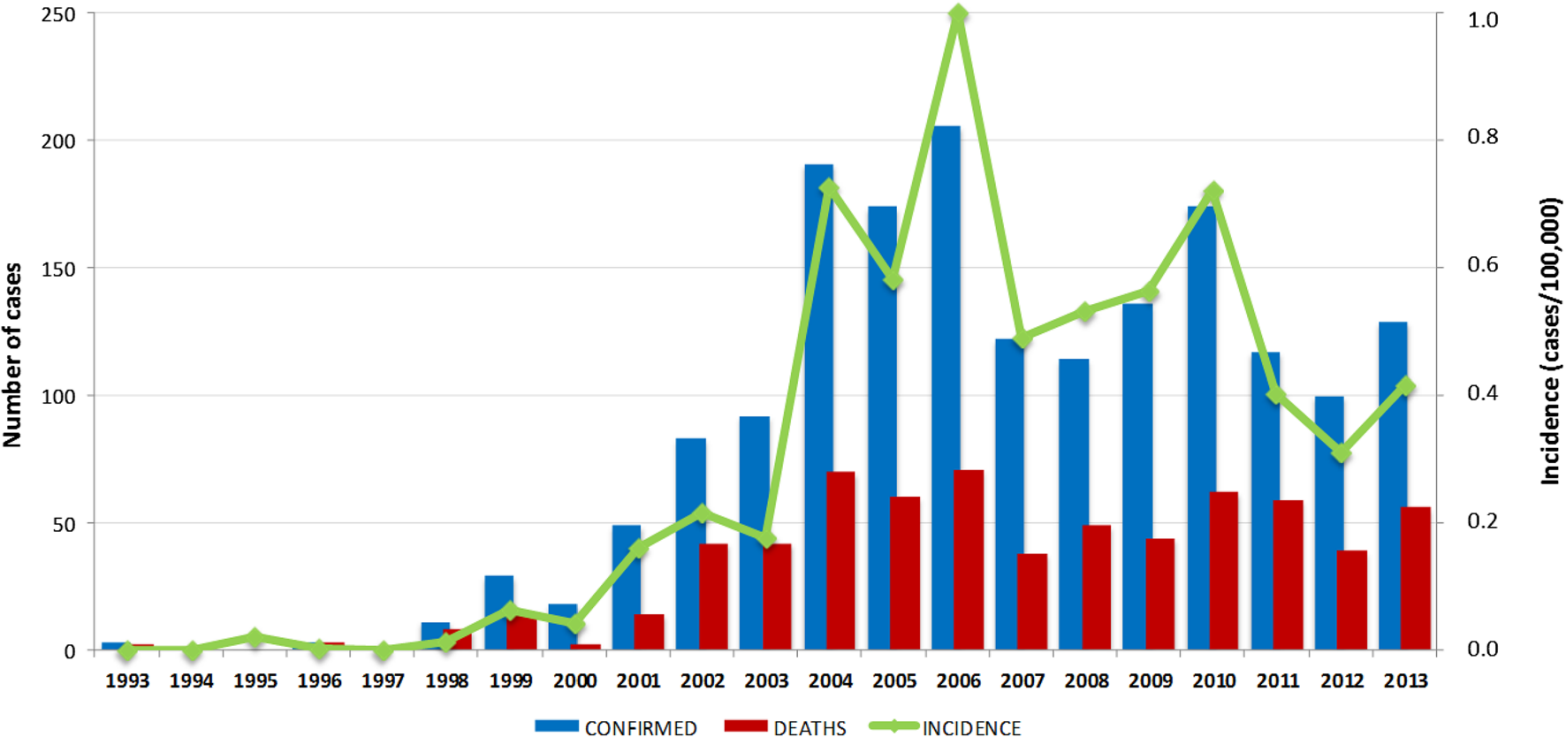
Number of cases of hantavirus, average incidence and number of deaths between 1993 and 2013 in Brazil

From 1993 to 2013, 1752 cases of hantavirus were registered in 16 Brazilian states. Most of cases of hantavirus were confirmed by laboratory tests (1637; 93.5 %); 79 (4.5 %) were confirmed by clinical and epidemiological criteria and 36 (2 %) were left blank in the forms. On average, 158 cases were registered per year, with a case-fatality rate of 38.5 % (Fig. 2). Most cases were registered in the states of Mato Grosso (285, 16.3 %), Santa Catarina (281, 16.0 %), Minas Gerais (233, 13.3 %), Paraná (205, 11.7 %), and São Paulo (190, 10.8 %). In total, hantavirus infections were registered in 464 municipalities. Santo Afonso and Campo Novo do Parecis, municipalities in the state of Mato Grosso, were found to have the highest incidences, at 15.7/100,000 and 13.4/100,000, respectively.

**Fig. 3:**
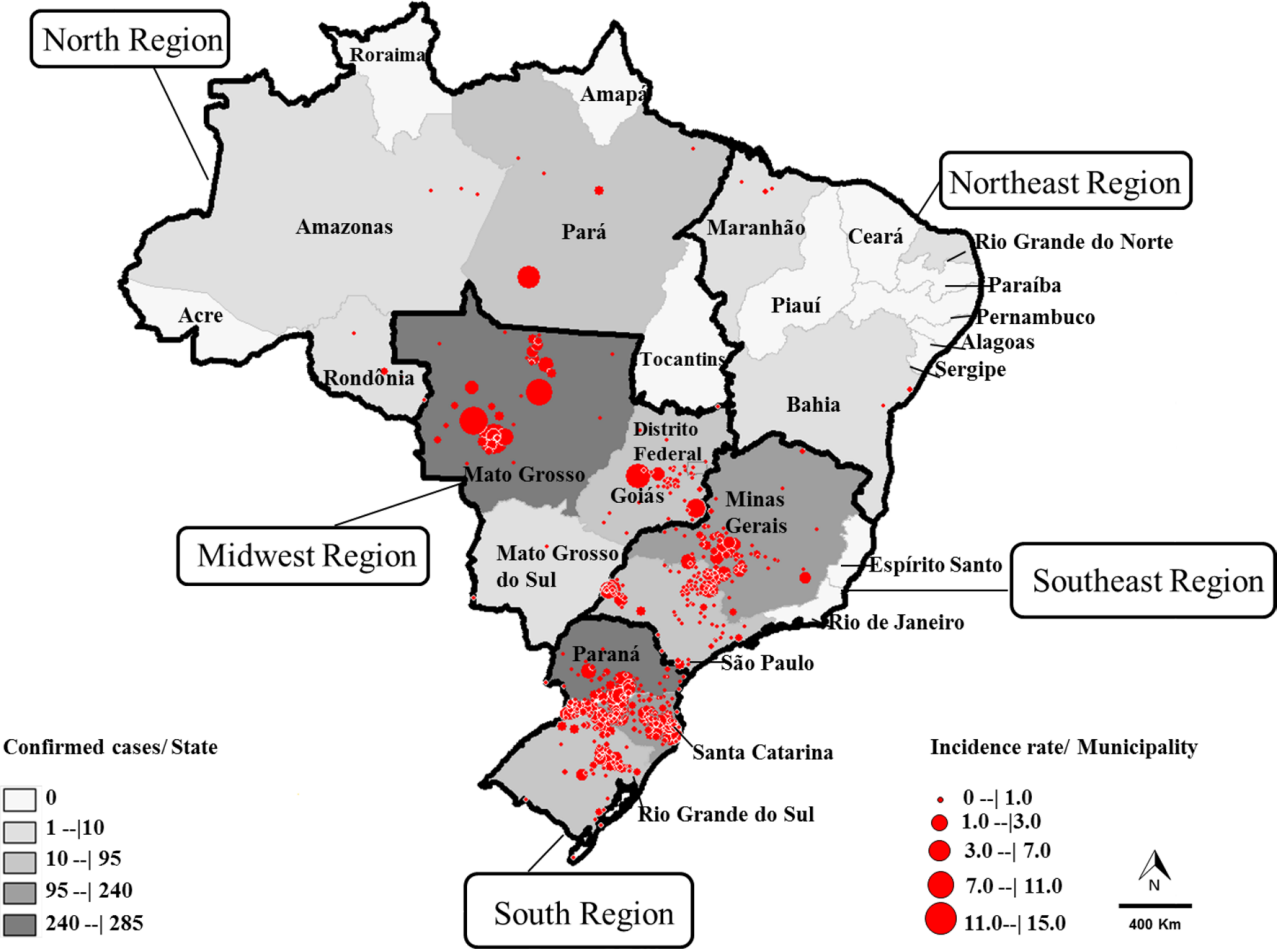
Number of cases of hantavirus per Brazilian state and incidence of hantavirus per municipality in Brazil from 1993 to 2013

As shown in Fig. 3, the highest incidence rates were observed in the states of Mato Grosso (0.57/100,000) and Santa Catarina (0.13), and the highest case-fatality rates were observed in the states of Rio Grande do Norte (100 %), São Paulo (54.2 %), Mato Grosso do Sul (50 %), Goiás (46.3 %), Pará (42.8 %), and Distrito Federal (41.1 %).

Epidemiological and demographic profiles of confirmed cases of hantavirus during the period under analysis are presented in Table 1. A higher incidence rate was observed in the midwestern region, where most of the people infected were adult white males from rural areas. However, higher fatality rates were observed among mixed-race females between 20 and 34 years of age.

**Table 1:** Results of an epidemiological and demographic descriptive study of confirmed cases of hantavirus in Brazil between 1993 and 2013

Epidemiological and demographic variable	Number of cases		Deaths	Case fatality rate	Incidence rate
	n	%	n	%	/100,000
Region of probable infection site					
North	95	3.5	40	42.1	0.34
Northeast	11	0.4	5	45.4	<0.01
Southeast	423	14.2	194	45.8	0.05
South	574	19.3	192	33.4	0.25
Midwest	472	15.9	184	38.9	0.90
Left blank or unknown	177	10.3	63	34.4	–
Infection zone					
Urban	151	8.6	60	39.7	–
Rural	1261	72.0	460	36.5	–
Periurban	110	6.3	48	42.7	–
Left blank or unknown	230	13.1	108	51.3	–
Gender					
Male	1338	76.4	503	37.6	0.04
Female	414	23.6	173	41.6	<0.01
Left blank	0	0.0	0	0.0	<0.01
Ethnic group					
White	1047	59.8	368	35.1	–
Black	91	5.2	40	44.0	–
Asian	18	1.0	4	22.2	–
Mixed race	363	20.7	165	45.5	–
Indigenous	38	2.2	3	7.9	–
Left blank	195	11.1	96	49.2	–
Age					
Less than 1 year	7	0.4	4	57.14	0.01
1–4 years	6	0.3	1	16.7	<0.01
5–9 years	20	1.1	7	35.0	0.01
10–14 years	43	2.5	16	37.2	0.02
15–19 years	91	5.2	58	63.7	0.03
20–34 years	426	24.3	282	66.2	0.04
35–49 years	662	37.8	205	31.0	0.09
50–64 years	363	20.7	92	25.3	0.09
65+	134	7.6	11	8.2	0.05

**Fig. 4:**
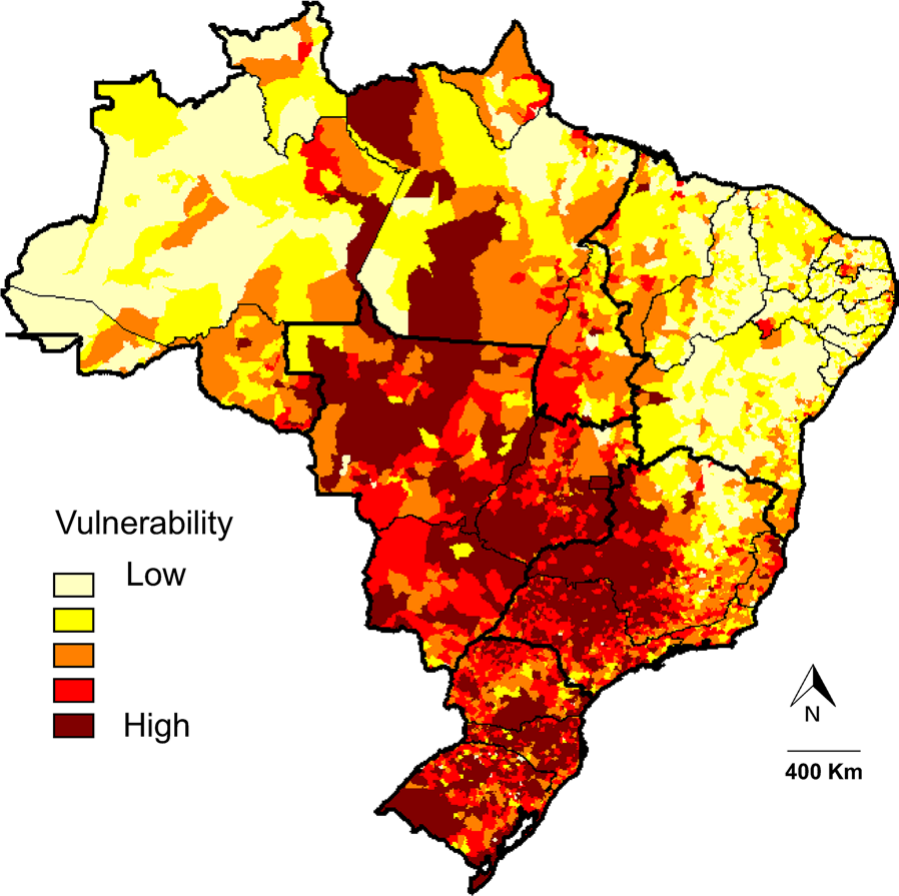
Classification of vulnerability to HCPS in Brazil based on multi-criteria decision analysis and epidemiological, demographic, and socioeconomic indicators. The map shows a chromatic scale representing areas of *higher* vulnerability in *dark red* and *lower* vulnerability in *light yellow*

Based on the MCDA, municipalities in southern, southeastern, and midwestern Brazil were classified as highly vulnerable (Fig. 4). Most of the municipalities in northern and northeastern Brazil were classified as having low vulnerability to HCPS.

## Discussion

Hantavirus infections have been registered all over Brazil over the last 20 years, but the incidence is higher in two states (Mato Grosso and Santa Catarina). Most of the most vulnerable municipalities were located in Midwest Brazil. The number of cases of hantavirus in Brazil over the last 20 years was twice as high as the number in the USA during the same period [27]. Moreover, the high case-fatality rates provide evidence of the virus’s impact on public health and underscore the need for a specific and reliable hantavirus infection diagnosis techniques in the country [28]. An HCPS treatment protocol has been recommended by the Brazilian Ministry of Health of Brazil [8], but the high case-fatality rates observed are associated with low clinical suspicion of hantavirus infections.

The results found herein indicate a large area of vulnerability that primarily encompasses southern, southeastern, and midwestern Brazil. Most municipalities in northern and northeastern Brazil were found to have lower vulnerability to HCPS. Agricultural activities and urban expansion toward forested areas may favor the spread of hantavirus, as observed by Santos [16]. The current study showed that the municipalities with greater vulnerability to occurrence of hantavirus had agriculture-based economies and many areas of urban expansion. These factors may promote interactions between rodents and humans. The municipalities with the highest incidence rates (Santo Afonso and Campo Novo do Parecis) are located in the in the state of Mato Grosso, which is within the midwestern region of the country and which represents a Cerrado-Amazon transition area. In recent years, widespread expansion of the agricultural frontier has occurred in these municipalities, which now have agriculture-based economies.

Hantavirus infections have recently been reported in municipalities within northern and northeastern Brazil [29, 30]. In the northeastern region, hantavirus infections have been reported in the states of Maranhão, Rio Grande do Norte, Bahia, and Ceará [31–34]. Interestingly, most of the municipalities in the state of Mato Grosso do Sul presented high vulnerability, a finding which is in contrast with the low HPCS incidence in this state. Because neighboring states have recorded several cases of hantavirus in recent years, it is likely that many cases go unreported in this state. Therefore, we recommend strengthened hantavirus surveillance in Mato Grosso do Sul.

Limitations of this study include the use of secondary data from a passive surveillance system implemented by the Brazilian Ministry of Health, as well as the inability to perform temporal patterning of the socioeconomic and demographic indicators obtained from 2006 to 2008. However, given the wide territorial range of Brazil and the increased incidence of HCPS since 2005, we believe that these limitations have not influenced the final results of the study. Future studies analyzing vulnerability to hantavirus infections should include data on mammal reservoirs. We did not consider host data, because few studies in the literature analyzed infection rates among rodent population in Brazil [35, 36]. However, a study analyzed the distribution of two main rodent reservoirs in the Atlantic rainforest and in the Brazilian Cerrado and estimated a broader area of risk of hantavirus transmission in the southeastern and southern regions of Brazil, an area which coincided with HCPS distribution [35].

MCDA is an innovative method that can be used to estimate vulnerability to tropical diseases. The PRADIN software was developed to help train technicians in the public sector and in non-governmental organizations, as well as researchers working with social indicators and public policy [25]. Its use in classifying vulnerability to disease can support surveillance and disease control programs. Stevens et al. [37] mapped the introduction and spread of H5N1 avian influenza virus in African countries and identified several vulnerable areas. Sarkar et al. [21] analyzed suitability for vector species and identified areas of high risk for Chagas disease in Texas. Multi-criteria techniques have also been used to identify which counties are at high risk of Chagas disease occurrence, even if the parasite has not yet been reported within their borders. The Brazilian Ministry of Health recommends MDCA for determining municipalities’ vulnerability to disease [38]. The Brazilian Program for Chagas Disease Control has also applied the MCDA to analyze municipalities’ vulnerability to this disease [21]. In Canada, MCDA has also been used in an innovative approach to zoonosis management, with promising results for Lyme disease [39].

Although most human infections by hantavirus have been registered in southern Brazil, a greater vulnerability to hantavirus was estimated in the midwestern region of the country. This disparity reflects the need for strengthened surveillance in areas where the disease has thus far gone unreported. These results may help guide more adequate surveillance activities and the prioritization of interventions the reduce frequency and severity of HCPS cases in Brazil.
